# Simple but long-lasting: A specimen imaging method applicable for small- and medium-sized herbaria

**DOI:** 10.3897/phytokeys.118.29434

**Published:** 2019-02-18

**Authors:** Atsuko Takano, Yasuhiko Horiuchi, Yu Fujimoto, Kouta Aoki, Hiromune Mitsuhashi, Akira Takahashi

**Affiliations:** 1 Museum of Nature and Human Activities, Hyogo, 6 chome, Yayoigaoka, Sanda, Hyogo 669-1546, Japan Museum of Nature and Human Activities Sanda Japan; 2 The Field, NPO corporation, Showa-dai 2-27-2, Takatsuki, Osaka 569-1041, Japan The Field, NPO Takatsuki Japan; 3 Department of Geography, Nara University, Misasagi 1500, Nara, Japan Nara University Nara Japan; 4 Graduate School of Culture and Information Science, Doshisha University, Mukokukan, 1-3 Tatara Miyakodani 1-3, Kyotanabe, 610-0394, Kyoto, Japan Doshisya University Kyotanabe Japan; 5 Institute of Natural and Environmental Science, University of Hyogo, 6 chome, Yayoigaoka, Sanda, Hyogo 669-1546, Japan University of Hyogo Sanda Japan

**Keywords:** Digitisation, Herbarium specimen, Imaging

## Abstract

Major international herbaria, natural history museums and universities have recently begun to digitise their collections to facilitate studies and improve access to collections. In Japan, more than 10 million herbarium specimens are housed in various universities/museums; however, only 1% of these have been digitised. In this paper, we describe a new method for imaging herbarium specimens that is applicable to local/small herbaria. It is safe, fast, simple and inexpensive, but also satisfies usage guidelines for minimum image quality and can produce digital files suitable for long-term storage and future post production. During an eight-month trial at the Museum of Nature and Human Activities, Hyogo, with three part-time workers using a custom-made copy stand and a mirrorless interchangeable lens camera with a large LED light bank system, we were able to image 73,180 herbarium specimens (571 per day on average), obtaining two RAW and two JPEG files for each specimen.

## Introduction

The digitisation of museum collections has recently become a major topic of discussion (e.g. Thiers et al. 2016, Le Bras et al. 2017, Barber et al. 2018, Sweeney et al. 2018). The Naturalis Biodiversity Center is in the process of digitising 37 million objects (https://science.naturalis.nl/en/collection/digitization/digitization/). The Finnish Museum of Natural History (Tabelberg et al. 2012) is running mass digitisation programmes for their entire collections. Furthermore, major herbaria around the world – such as Harvard University (A, GH, NEBC), the Royal Botanic Garden, Edinburgh (E), the Royal Botanic Gardens, Kew (K), the French Muséum national d’histoire naturelle vascular plant herbarium (P, Le Bras et al. 2017) and the Meise Botanic Garden (BR, De Smedt et al. 2018) – have also conducted digitisation. China (the Chinese Virtual herbarium [http://www.cvh.ac.cn/]) and the United States (Integrated Digitized Biocollections [iDigBio]; https://www.idigbio.org/) promote specimen digitisation as national projects. A list of the major digital herbaria in the world was compiled and is shown in Table 1.

**Table 1.** A list of the major virtual herbarium in the world.

Location	Herbarium	URL
**North America**		
U.S.A	Atrium Biodiversity Information System	http://atrium.andsamazon.org/digital_herbarium.php
	Hervard University Herbaria	http://kiki.huh.harvard.edu/databases/specimen_index.html
	Missouri Botanical Garden	http://www.tropicos.org/
	Field Museum, Chicago	http://emuweb.fieldmuseum.org/botany/detailed.php
	Smithonian Institution National Museum of Natural History	https://collections.nmnh.si.edu/search/botany/?ti=3
	New York Botanical Garden	http://sweetgum.nybg.org/science/vh/
	University of Florida Herbarium	http://www.flmnh.ufl.edu/herbarium/cat/imagesearch.asp
**South America**		
Colombia	Universidad Nacional de Colombia	http://www.biovirtual.unal.edu.co/es/
Brasil	Centro de Referência em Informação Ambiental CRIA	http://splink.cria.org.br/
**Europe**		
U.K.	Natural History Museum, London	http://data.nhm.ac.uk/dataset/collection-specimens
	Royal Botanic Gardens, Kew	http://apps.kew.org/herbcat/navigator.do
	Royal Botanic Gardens, Edinburgh	http://data.rbge.org.uk/search/herbarium/
	Botanical Society of the British Isles	http://herbariaunited.org
Finland	Finnish museum of natural History	https://www.luomus.fi/en/botanical-and-mycological-collections
Bergium	Botanic Garden Meise	http://www.br.fgov.be/RESEARCH/COLLECTIONS/HERBARIUM/advancedsearch.php
France	Museum National D'histoire Naturelle	https://science.mnhn.fr/institution/mnhn/collection/p/item/search/form?lang=en_US
Denmark	Aarhus University	http://www.aubot.dk/search_form.php
Austria	Herbarium WU, University of Vienna	https://herbarium.univie.ac.at/database/search.php
Switzerland	Zürcher Herbarien, University of Zurich	https://www.herbarien.uzh.ch/en.html
Germany	Botanischer Garten und Botanishes Museum Berlin	http://ww2.bgbm.org/herbarium/default.cfm
Croatia	University of Zagreb	http://herbarium.agr.hr/search.html
**Oceania**		
	The Australasian Virtual Herbarium	http://avh.chah.org.au/
New Zealand	New Zealand national herbarium network	http://www.virtualherbarium.org.nz
**Asia**		
China	Chinese Virtual Herbarium	http://www.cvh.org.cn/
	Chinese Academy of Sciences Inst. Botany -Beijing	http://pe.ibcas.ac.cn/herbs/herbariumsearch.aspx
Thailand	Thai Forest Herbarium type specimen database	http://www.dnp.go.th/botany/bkfmain.aspx
Japan	National Museum of Nature and Science(TNS) Type specimen Database	http://www.type.kahaku.go.jp/TypeDB/
	Makino Herbarium Type Specimen Image Database	http://ameba.i.hosei.ac.jp/BIDP/MakinoCD/makino/html_j/index0.html
	Shimane University ditital herbarium	http://tayousei.life.shimane-u.ac.jp/harbarium/
	The University of Tokyo, type collection database	http://umdb.um.u-tokyo.ac.jp/DShokubu/herbarium/en_ver2/index.php

Compared to the aforementioned countries, Japan is rather behind (Ogawa 2008, Moriguchi et al. 2012, Akihiro et al. 2018). About 10 million herbarium specimens are housed in universities and museums in Japan (The Unions of the Japanese Societies for Systematic Biology 2015) and half of these are deposited at the three largest herbaria: The University of Tokyo (TI) with ca. 1.8 million specimens, the National Museum of Science and Nature (TNS) with ca. 1.7 million specimens and the Kyoto UniversityMuseum (KYO) with ca. 1.2 million, while the rest are dispersed in smaller, local herbaria. At present, 20% (ca. 2 million) of the specimen data in Japan have been deposited in the Global Biodiversity Information Facility (G-BIF) (https://www.gbif.org/) via J-BIF, the Japan node of G-BIF (http://www.gbif.jp/v2/). This facility provides text without images. Some herbaria in Japan – such as TI (http://umdb.um.u-tokyo.ac.jp/DShokubu/herbarium/en_ver2/index.php), TNS (http://www.type.kahaku.go.jp/TypeDB/) and the Metropolitan University of Tokyo (MAK) (http://ameba.i.hosei.ac.jp/BIDP/MakinoCD/makino/html_e/index0.html) – have published databases of their herbarium collections with specimen images. However, these are limited to specimen type collections or specimens from certain areas (e.g. Nepal and oceanic Islands). The largest herbarium database in Japan with specimen images is the Shimane University virtual herbarium (http://tayousei.life.shimane-u.ac.jp/harbarium/) (Akihiro et al. 2018). Currently, ca. 100,000 specimen images from the Tottori Prefecture Museum (TRPM), the Shimane Nature Museum of Mt. Sanbe Sahimel, the Rikuzentakata City Museum and the Herbarium of Faculty of Symbiotic Systems Science at Fukushima University (FKSE) are available online.

Nelson et al. (2015) described three widely used imaging station alternatives for herbarium digitisation: (1) A copy stand with fluorescent lighting, (2) a light box with internal lighting and (3) an inverted flatbed scanner. In the present study, we explored another method and developed a custom-made digitisation system for herbarium specimens using a mirrorless interchangeable-lens camera (MILC) and a large bank light system, even though inverted flatbed scanners are most commonly used in Japan (e.g. Suh et al.2006, Ogawa 2008, Moriguchi et al. 2012, Akihiro et al. 2018). There were two reasons for using a digital camera: firstly, it is easier to take multiple images of one specimen in various forms using a camera. For security in the long-term storage of specimen images, it is better to have files of multiple kinds. The long term preservation of digital images is discussed in terms of file formats (Digital Preservation Handbook, 2^nd^ edition 2018). Well-known formats are PNG, TIFF, JPEG and JPEG 2000, whose specifications are freely available to users. Although JPEG is the common file format amongst these, it involves using non-invertible compression of the image. Due to this, TIFF is preferred in digital archiving projects, but there are currently no reasonably priced digital cameras able to save TIFF directly. Therefore, we acquired lossless RAW images, which can be converted to TIFF images in parallel with JPEG images. In contrast, flatbed scanners can obtain only one image file per scan and one scan takes up to several minutes to obtain sufficient image quality. Therefore, if we want to obtain multiple images in multiple formats per specimen using a scanner, the time taken will increase accordingly. Secondly, the thickness of specimens does not affect digital camera images. Bulky specimens (e.g. conifer cones and aroid bulbs) are difficult to scan but pose no problem for cameras.

The system we have developed is simple and inexpensive, requires minimal space, could be managed by part-time workers and makes it possible to easily obtain multiple standardised digital files of several kinds. Using a digital camera has often been avoided because it is thought to be extremely difficult to provide sufficient lighting for quality specimen imaging (Suh et al. 2006). We overcame this problem by adopting a light-bank system using LED light. This system is suitable for small and medium-sized herbaria where staff, space and budget are limited, as well as for larger herbaria with larger numbers of staff members and stations. It may also be applied when digitising other kinds of collections, including entomological, mineralogical and fossil collections.

## Materials and methods

### Target collections

As our target for digitisation, we chose the Shoei Junior College (Higashinada-ward, Kobe City, Hyogo, Japan) herbarium collection, which was previously known as SHO and is currently one of the collections at the Museum of Nature and Human Activities, Hyogo (HYO). The collection consists of ca. 250,000 specimens of vascular plants. SHO has a history of more than 80 years and, along with KYO, it is the most well-known herbarium in western Japan. Many taxonomists have visited SHO, examined the specimens and cited the SHO collection in their papers. Upon the retirement of two taxonomists from the college in 2012, the collection was donated to HYO. Due to current storage space limitations, 95% of the SHO herbarium sheets are kept separate from the main HYO collection in boxes. We set out to achieve the digitalisation of the collection by 2020 by first creating a searchable database to enhance the accessibility of the collection.

### Imaging policies

To complete digitisation within a limited time and budget, we decided to use the minimum acceptable quality point (MAQP) for the obtained images (Fujimoto and Horiuchi 2016, Fujimoto 2017) before developing equipment. Our terms of reference were as follows:

1 Images should be usable and suitable for long-term storage. It should be noted that capturing and preserving high-quality specimen images offers opportunities to take advantage of future improvements in image analysis (La Salle et al. 2009), optical character recognition (OCR) (Haston et al. 2012), natural language processing, handwriting analysis and data-mining technologies (Nelson et al. 2012).

2 Images should have enough resolution to withstand expansion up to 150% on a display monitor and be capable of withstanding life-size, high-definition printing.

3 Images should have applicable OCR for data transcription from the specimen label. To increase the accuracy of the OCR output, images obtained should have sharp margins and be flat with minimal distortion.

4 Imaging should be finished within two to three years using the same hardware (camera and lens) to keep the quality of all images consistent. Hardware lifespan is generally in the range of three to five years; that of a digital camera may be shorter.

### Component selection for the imaging station

Digital camera and lens choice

An MILC was selected for our imaging system. They are smaller, lighter and experience fewer vibrations from camera shake and shutter shock than digital single-lens reflex (DSLR) cameras. The lighter body made it easier for us to design a custom-made copy stand and the decreased susceptibility to lens aberrations is better for future OCR image use. After some trial and error, we chose the SONY α6300 (ILCE6300), APS-C sensor, digital e-mount camera and an FE 35 mm F.2.8 ultra- compact wide-angle lens for the Sony e-mount full frame (Samyang Optics SYIO35AF-E 35 mm F/2.8). Specimen images obtained using this combination of camera and lens are 5100×3500 pixels or ca. 25 MB in size.

Custom-made copy stand with LED lighting system

**Figure 1.**
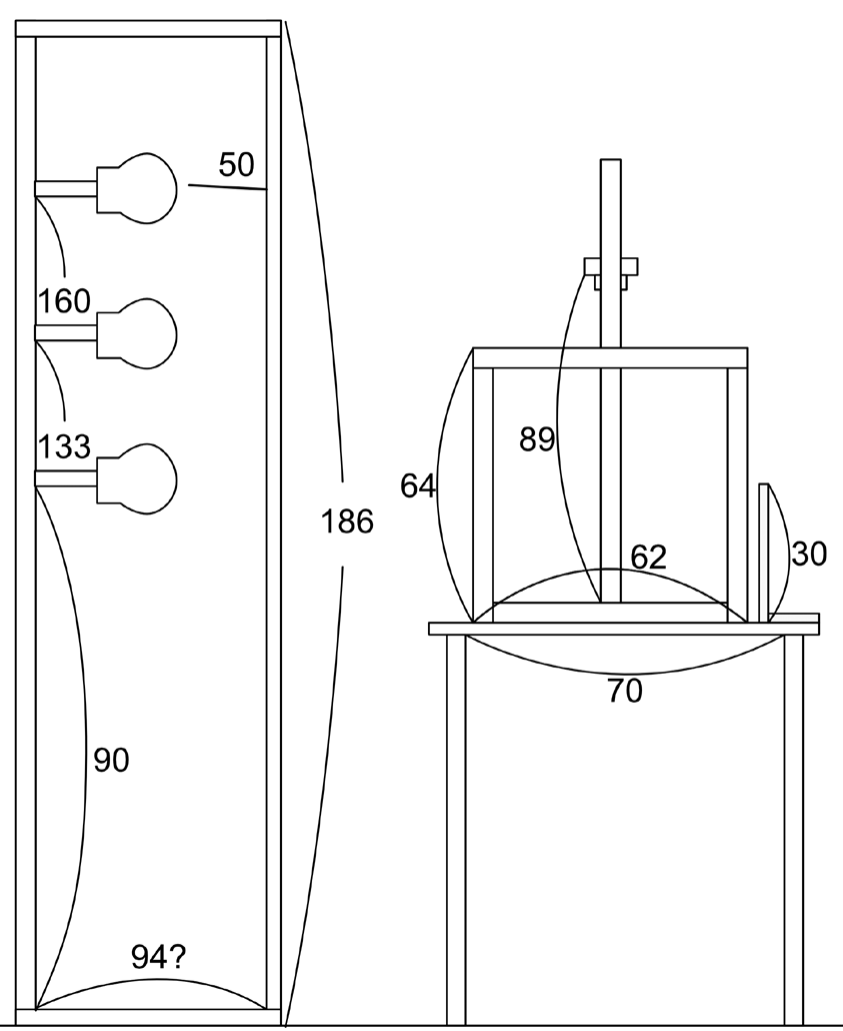
Side view of the imaging equipment we developed. The numbers show the size of each part (cm).

**Figure 2.**
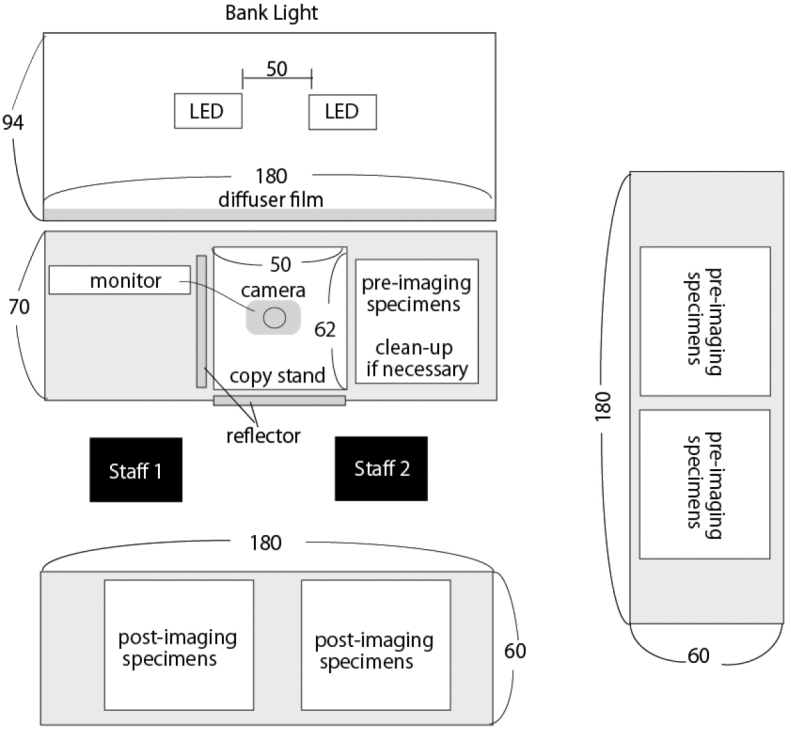
Birds-eye-view of imaging equipment. Numbers indicate size (cm).

**Figure 3.**
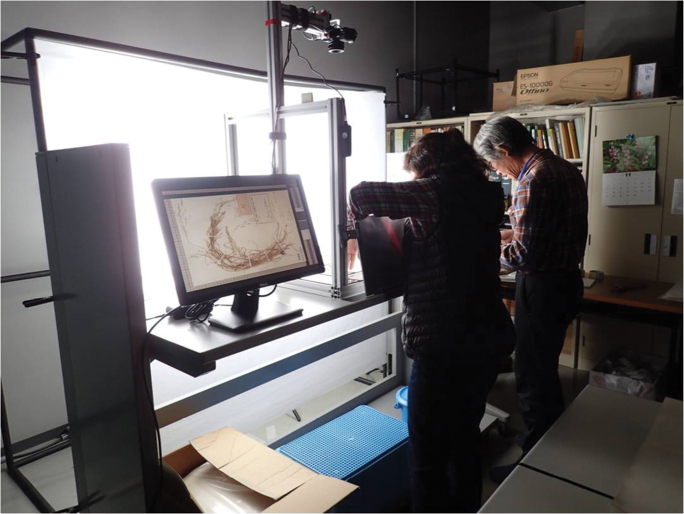
Employees working on the imaging system.

LED light is the only light source that does not generate heat and that offers efficient electric lighting with control over the intensity and wavelength, allowing for the reduction of UV light. LED light is thus beneficial for both specimens and workers’ health. To record the colour of herbarium specimens precisely at reasonable cost, LED light with a high colour rendering property was selected (039 SH50 Pro-S LED Lamp, China) and diffuser film was also chosen to be durable enough for frequent changes over a long period (Savage Translum^TM^, U.S.A). A large light bank system was designed to apply sufficient light above the specimen in a manner similar to a skylight (Figs 1–3). This involved using two light stands, each bearing three of the LED lights mentioned above, located 50 cm apart from each other and 50 cm behind the diffuser film, to provide sufficient light intensity and to obtain a sharp and clear image of the plants on the sheet (Figs 1–3).

We constructed our own copy stand for imaging specimens because ready-made copy stands are too large, heavy, complicated and expensive. As we chose a lighter MILC, the stand did not have to be as robust to avoid camera shake. The design drawing is provided in Fig. 1. We adapted a lightweight aluminium frame structure with excellent durability and practical use strength for reducing camera shake (Yuki corp., Aichi Japan). On the copy stand, we put a mark to indicate where to place herbarium sheets and the GIN-ICHI Silk Gray Card.

Space required

For the work production line, we needed space for the light bank, pre-imaging specimens, copy stand and post-imaging specimens and a monitor to check imaging (Fig. 3). A total of ca. 27 m^2^ was required for this imaging system. Additional space for specimen repair, a laptop and an NAS server for saving images were also needed.

Staff training

Three digitisers were hired through public advertisement. None of them was a photographer and two had no experience with handling herbarium specimens. We trained them on how to treat specimens and the specimen imaging workflow in one day. After that, they worked in alternating pairs. One of them treated (if necessary) and moved the target specimen from the pre-position to the copy stand and the other operated the remote-control shutter and checked the image in the monitor (Fig. 3). Protocols are detailed below.

### Imaging protocol

***Pre-imaging***

1) Take target specimens from storage

2) Remove specimen from the genus cover

3) Clean-up and/or repair specimens (if necessary)

4) Apply a barcode to the specimen sheet

5) Place it on the copy stand in accordance with the guide

***Imaging***

After confirmation that all objects are clean and in accordance with the pre-imaging settings above, the shutter is released to capture the image(s) with the following conditions.

1) Fixed camera working distance (ca. 89 cm)

2) Shutter speed 1/50-60, ISO 100

3) White balance measurement using a GIN-ICHI Silk Gray Card

We took multiple photos to obtain two 48-bit RAW and two JPEG files for each specimen.

***Post-imaging***

1) Check image quality (focus, exposure etc.)

2) Remove specimen from the copy stand

3) Clean-up dust on copy stand (if necessary)

4) Clean-up and/or repair specimens (if necessary)

5) Return specimen to the original cover

6) Apply mark on the genus cover to indicate completion of imaging

7) Place them back into storage after cold fumigation (−90 °C, 10 h)

***Save files to the NAS server (at the end of the day)***

1) Create a new folder named according to the date in the server

2) Put all images collected on that day into the named folder

3) Copy these files to another external HDD

## Results

**Figure 4.**
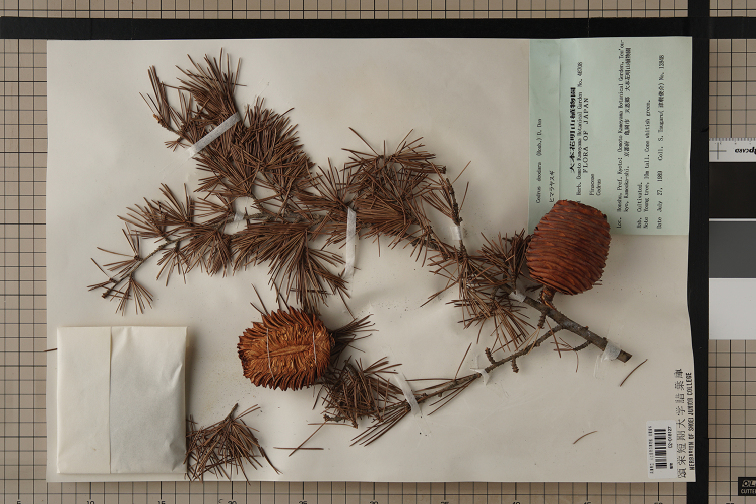
An image of herbarium specimens taken using imaging method described in this study (*Cedrusdeodara* (Roxb.)D.Don, HYOID: C2-018127).

Specimen images, obtained using our method, are of a quality suitable for OCR output (Fig. 4). All procedures, from pre-digitisation curation to storage and archiving of images, were performed by two part-time workers between 9 am and 5 pm each day. The speed of imaging depended on how many specimens needed conservation or clean-up before and/or after imaging. Most herbaceous specimens, especially Poaceae, Cyperaceae and Saxifragaceae, contained substantial amounts of sand or dry mud amongst their roots and took time to clean-up before imaging. Therefore, we sometimes obtained only 1,200 images (300 specimens) per day. In contrast, for woody or large herbaceous specimens without roots (and associated soil), imaging ran smoothly and we could obtain up to 4,000 images (1,000 specimens) per day. From the start of guidance and training on 10 Nov 2017 to 4 July 2018, a total of 73,180 specimens were imaged and stored as RAW and JPEG files.

## Discussion

Our imaging system minimises both initial costs and space requirements using a custom-made copy stand alongside an MILC and other ordinary devices. The imaging system described here costs ca. 314,000 JPY in total (US $2,778), including 170,000 JPY for the MILC camera, lens, adaptor and monitor, 90,000 for the bank light system (incl. LED lights, ramp folders, diffusion film etc.) and 54,000 for the camera stand. The development of new technologies has afforded us the use of LED or OLED lighting at a low cost. Using a large LED bank light system, we were able to obtain images that were rich in contrast and gradation without high resolution. In addition, the working space was minimised by putting a copy stand and before and after imaging of specimens in line on the same desk, making the workflow compact and smooth. This system is easy to operate, making it simple to train workers. It provides the ability to obtain multiple 48-bit RAW and JPEG files for each specimen. Obtaining multiple files and multiple kinds of digital files is important for long-term storage.

### Additional costs

After three months, during which 40,000 specimens were imaged, a digital camera broke. Repair was possible and free under warranty, but image processing was stopped during the repair period. A remote-control shutter also broke twice during the trial period. Therefore, it would be advantageous to have spare equipment, if possible.

### Future tasks

Images were stored under a default name on the NAS server day by day. We applied a barcode before imaging that bore a unique ID at our museum and we needed to rename the image files to this unique ID number using a barcode reader. Developing and running simple programmes to rename images will be our next goal. Furthermore, to publish searchable specimen images on the web, transcription of label data (Scientific name, Loc. Date etc.) is necessary. OCR has begun to be used to transcribe data from specimen labels over the last ten years (Moen et al. 2008, Heidorn and Wei 2008). There are some OCR-based semi-automatic label information extraction systems, including SALIX (Barber et al. 2013) and HERBIS (Beaman et al. 2006). We are also working to develop a semi-automatic label information extraction programme (Aoki et al. in prep.) that uses Dlib [1] (King 2009) to detect the label area and annotation card area for cropping; these are then used to run the Tesseract open source OCR engine ver. 3.0.4. (Smith 2007). Using this system, label information has been successfully extracted from 1584 of 1970 specimen images (80%).

Digitisation of herbarium specimens benefits both curators and stakeholders: for stakeholders, it becomes possible to access a digital voucher for each specimen remotely via the internet and, for curators, it reduces the need for specimen handling and makes semi-automated label data extraction by OCR possible. Further, crowdsourcing the manual data entry of specimen labels can be considered, given remote access to specimen images. Specimen processing, from mounting until manual data entry, can be facilitated, updated and automated wherever possible as technology develops. The imaging of herbarium specimens is the first important step in this process.

## Conclusion

We developed a new digital imaging system for herbaria that takes up little space, minimises costs, is simple to use and quickly creates data that can be archived long-term and we provide here a step-by-step guide to create the system. We hope our imaging system will facilitate the digitisation of small- and medium- sized herbaria where investment possibilities are limited.
